# Genetic manipulation of *Methanosarcina* spp.

**DOI:** 10.3389/fmicb.2012.00259

**Published:** 2012-07-24

**Authors:** Petra R. A. Kohler, William W. Metcalf

**Affiliations:** Department of Microbiology, B103 Chemical and Life Science Laboratory, University of Illinois at Urbana-ChampaignUrbana, IL, USA

**Keywords:** *Methanosarcina*, genetic manipulation, mutagenesis, markerless deletion, genetypic complementation, gene expression

## Abstract

The discovery of the third domain of life, the Archaea, is one of the most exciting findings of the last century. These remarkable prokaryotes are well known for their adaptations to extreme environments; however, Archaea have also conquered moderate environments. Many of the archaeal biochemical processes, such as methane production, are unique in nature and therefore of great scientific interest. Although formerly restricted to biochemical and physiological studies, sophisticated systems for genetic manipulation have been developed during the last two decades for methanogenic archaea, halophilic archaea and thermophilic, sulfur-metabolizing archaea. The availability of these tools has allowed for more complete studies of archaeal physiology and metabolism and most importantly provides the basis for the investigation of gene expression, regulation and function. In this review we provide an overview of methods for genetic manipulation of *Methanosarcina* spp., a group of methanogenic archaea that are key players in the global carbon cycle and which can be found in a variety of anaerobic environments.

## Introduction

Life on earth, as currently perceived by mankind, is encompassed in three major domains Archaea, Eukarya and Bacteria. The recognition of Archaea as a distinct phylogenetic lineage is a fairly recent discovery (Woese et al., 1990). Initially it was assumed that Archaea were strictly anaerobic and mostly extremophiles, inhabiting environmental niches hostile to most other organisms, such as submarine volcanic vents, solfataric hot springs, or soda lakes. It is known today that Archaea are ubiquitous, represent a significant portion of the global biomass and play important roles in global ecosystems and biochemical cycling (Delong and Pace, 2001; Jarrell et al., 2011).

Archaea share metabolic and physiologic features with Eukarya and Bacteria, but they are also unique in many ways (Jarrell et al., 2011; Jun et al., 2011; White, 2011). In many cases Archaea are uniquely positioned to carry out biochemical reactions that are of significant interest for industrial and biotechnological applications. Prominent examples are: methanogenesis, the reduction of CO_2_ to methane; or the application of Bacteriorhodopsin, a light driven proton pump, in solar cells and radiation sensors (Thavasi et al., 2009; Ahmadi and Yeow, 2011; De Vrieze et al., 2012).

Biochemical, structural and physiological studies have provided significant insight into the third domain of life over the last three decades (Cavicchioli, 2011). However, our understanding of Archaea still lags behind our knowledge of Eukarya and Bacteria. From a scientific and biotechnological point of view this gap in knowledge needs to be filled urgently. An important step toward achieving this goal was the development of systems for genetic manipulation of members of the methanogenic archaea, the halophilic archaea and the thermophilic, sulfur-metabolizing archaea (Rother and Metcalf, 2005; Buan et al., 2011; Leigh et al., 2011).

The study of methanogenic Archaea has a long-standing history because of their central role in the global carbon cycle and their potential application in biofuel production (Fox et al., 1977; Jarrell et al., 2011; De Vrieze et al., 2012). Methanogenic metabolism converts a limited number of one-carbon (C-1) compounds and acetic acid to methane through a series of coenzyme-bound intermediates in a process that drives the generation of ATP (Thauer et al., 2008). Methanogenic Archaea are represented in five orders, the *Methanococcales*, the *Methanosarcinales*, the *Methanobacteriales*, the *Methanomicrobiales* and the *Methanopyrales* (Liu and Whitman, 2008). Genetic systems are available for organisms in the first two orders (Rother and Metcalf, 2005; Leigh et al., 2011). Three classes of methanogens have been proposed based on physiological, biochemical and genomic traits (Anderson et al., 2009). Class I methanogens, *Methanococcales, Methanobacteriales* and *Methanopyrales*, and class II methanogens, *Methanomicrobiales*, are usually hydrogenotrophic; they use *H*_2_/*CO*_2_ and sometimes formate as substrates for methanogenesis (Anderson et al., 2009). Class III methanogens, *Methanosarcinales* metabolize a variety of substrates, *H*_2_/*CO*_2_, C-1 compounds, such as methylamines, methylsulfides or methanol, and acetate in four different methanogenic pathways (Thauer et al., 2008; Anderson et al., 2009). Three organisms, *Methanosarcina bakeri* Fusaro and *Methanosarcina acetivorans* C2A and *Methanosarcina mazei* Gö1 are well established in methanogenesis research and the complete genome sequences of all three organisms are publicly available. The major difference between these organisms lies within their ability to utilize methanogenic substrates, *M. bakeri* Fusaro and *M. mazei* Gö1 use all known substrates, whereas *M. acetivorans* C2A lacks the ability to grow on *H*_2_ and *CO*_2_ (Thauer et al., 2008; Guss et al., 2009; Kulkarni et al., 2009).

In the past 15 years, a number of techniques that allow the study of gene function *in vivo* have been developed for these *Methanosarcina* species. This review focuses on the genetic manipulation of *Methanosarcina* spp. and provides an overview about established methods including, random and targeted mutagenesis, complementation, and reporter gene fusions.

## Transcription, translation, and DNA repair in archaea

The transcription, translation, and DNA repair systems play an important role during genetic manipulation and therefore need to be considered for method development. The proteins involved in DNA repair facilitate the incorporation of cloned DNA into the genome of the target organisms, while the expression of cloned genes and selective markers is driven by the transcription and translation system.

Like bacteria, known Archaea store their genetic information on a circular chromosome and in many cases on additional plasmids (Keeling et al., 1994; Koonin and Wolf, 2008). In addition, transcriptional units often comprise operons (Brown et al., 1989). Nevertheless, the basal archaeal transcription apparatus including the 11- to 13-subunit DNA-dependent RNA Polymerase (RNAP) is closely related to the eukaryotic Pol II system and some rRNAs and tRNAs were found to be characteristic for Archaea (Woese et al., 1978; Jun et al., 2011).

The archaeal promoter is very similar to that of eukaryotes and includes a TATA box element located approximately 30 bp upstream of the transcriptional start (Hausner et al., 1991; Jun et al., 2011). At least two factors, homologues of the eukaryotic TATA-binding protein (TBP) and Transcription Factor II B (TFIIB), are required for transcription initiation. TFIIB interacts with the B recognition element (BRE), a purine rich sequence located directly upstream of the TATA-box (Jun et al., 2011). The majority of the transcriptional regulators identified to date are homologous to known bacterial regulators and transcriptional regulation generally seems to follow the bacterial model. This is especially evident from the mechanisms of actions described for transcriptional repressors that bind DNA close to the promoter and either occlude the TATA box and the BRE element or inhibit the recruitment of the RNAP (Bell, 2005; Jun et al., 2011; Malys and McCarthy, 2011). However, archaeal histones were shown to also be involved in transcription regulation similar to what is known for Eukaryotes (Reeve, 2003; Jun et al., 2011).

Archaeal mRNAs do not posses a 5′ cap structure and long poly-A tails (Brown and Reeve, 1985, 1986; Hennigan and Reeve, 1994). Translation can occur in a Shine-Dalgarno-dependent or -independent fashion; mRNAs lacking a Shine-Dalgarno sequence are either leaderless or are led by a 5′ untranslated region (Dennis, 1997; Malys and McCarthy, 2011).

DNA repair is achieved through double stranded break repair and homologous recombination. Mechanistically homologous recombination follows the eukaryotic model and the key enzymes involved, Mre11, RadA, and Rad50, are also found in Eukaryotes (White, 2011).

## Cultivation of *methanosarcina* spp.

Successfully working with any organism requires the ability to satisfy their nutritional and environmental needs. *Methanosarcina* spp. and methanogens, in general, are very sensitive to oxygen. All experimental manipulations and the cultivation of strains must be performed under strict anaerobic conditions. Media are commonly bicarbonate buffered minimal media with a pH from 6.8 to 7.0 and must have a redox potential of at least –300 mV to keep *Methanosarcina* metabolically functional (Balch et al., 1979; Sowers et al., 1993; Metcalf et al., 1998).

## DNA delivery, exchangeable promoters, selectable, and counterselectable markers

The successful genetic manipulation of any organism depends on four basic requirements: (1) The ability to grow clonal colonies from single cells, (2) a DNA delivery system, (3) promoters for the expression of cloned genes, and (4) selectable genetic markers.

*Methanosarcina* often grow in multicellular packets containing from a few, up to tens of thousands of cells. Aggregates of *Methanosarcina* cells are encapsulated by methanochondroitin, a positively charged exopolysaccharide composed of *N*-acetyl-D-galactosamine, galacturonic- and glucuronic-acid that interacts with the S-layer, a proteinaceous matrix that is found immediately adjacent to the cell membrane (Kreisl and Kandler, 1986; Ellen et al., 2010). Cell aggregates represent a physical barrier for exogenous DNA and therefore impede genetic manipulation. The production of methanochondroitin seems to be environmentally regulated and it is not produced under conditions of high osmolarity, resulting in unicellular growth (Sowers et al., 1993). Based on this discovery high salt media were developed that allow the growth of clonal populations from single cells and render *Methanosarcina* more easily accessible for genetic manipulations (Sowers et al., 1993).

Liposome- and polyethylene glycol (PEG)-mediated transformation methods have been developed for *Methanosarcina* (Metcalf et al., 1997; Oelgeschlager and Rother, 2009). The former achieves high transformation frequencies, up to 2 × 10^8^ transformants per μ g DNA, representing about 20% of the CFU for *M. acetivorans*. Frequencies in other *Methanosarcina* spp. are slightly lower but still sufficient for most purposes (Metcalf et al., 1997; Ehlers et al., 2005).

Selectable or testable phenotypes are mandatory to monitor DNA up-take or exchange. The basic requirement, to achieve the establishment of a tractable phenotype through a selectable or counterselectable marker, is a reliable gene expression system. Two exchangeable transcription systems are routinely used for the expression of cloned genes in *Methanosarcina* spp. Both systems comprise a strong constitutive promoter, p*mcr* or p*mcrB*, that drives the transcription of the methyl-reductase operons in *Methanococcus voltae* and *Methanosarcina barkeri* Fusaro, respectively, and its corresponding ribosomal binding sites (RBS) and transcriptional terminators (Metcalf et al., 1997; Zhang et al., 2000).

Two antibiotic resistance markers have been developed for *Methanosarcina*. Resistance to the protein synthesis inhibitor Puromycin is easily achievable, by introducing the *pac* (puromycin transacetylase) gene from *Streptomyces alboniger* controlled by p*mcr* (Gernhardt et al., 1990; Metcalf et al., 1997). A second selectable marker, that codes for resistance to pseudomonic acid was created by mutagenesis of the isoleucyl-tRNA synthetase gene (*ileS12*) from *M. barkeri* Fusaro (Boccazzi et al., 2000).

A counterselectable marker system for the construction of *M. acetivorans* C2A and *M. barkeri* Fusaro mutants was developed based on the deletion of the *hpt* gene, encoding a hypoxanthine phosphoribosyltransferase (Pritchett et al., 2004; Guss et al., 2008). The Hpt protein is part of the purine salvage pathway and catalyzes the phosphorylation of various purines to the corresponding monophosphate. Toxic purine analogs like 8-aza-2,6-diaminopurine (8ADP) also serve as substrate for Hpt. Incorporation of these toxic bases into DNA can be lethal (Bowen and Whitman, 1987; Bowen et al., 1996). A *M. acetivorans* C2A Δ*hpt* strain is approximately 35-fold more resistant to 8ADP than the wild type; 8ADP sensitivity is restored upon reintroduction of the *hpt* gene. This phenotype has proven useful for the creation of unmarked deletion mutants and therefore a series of Δ*hpt* parental strains were constructed (see below; Pritchett et al., 2004; Guss et al., 2008).

## Shuttle vectors

A series of autonomously replicating *Escherichia coli*/*Methanosarcina* shuttle vectors have been developed based on the native *M. acetivorans* pC2A plasmid that is present in about six copies per cell (Sowers and Gunsalus, 1988; Metcalf et al., 1997). These constructs contain a *pac* cassette for selection and the pC2A replicon for replication in *Methanosarcina*. The plasmids can be used for a variety of *Methanosarcina* spp. The β-lactamase gene enables selection and *ori*R6Kγ allows replication in *E. coli*. The variety of plasmids provide different multi cloning sites and some include the *lacZ* α gene for blue and white screening to allow facile identification of recombinant plasmids in *E. coli* (Metcalf et al., 1997).

The shuttle plasmid vectors gave rise to a series of molecular tools that make forward and reverse genetics possible in *Methanosarcina*. All plasmids for the genetic manipulation of *Methanosarcina* are designed to be maintained in appropriate *E. coli* hosts. Ampicillin- (*bla*), chloramphenicol- (*cat*) or kanamycin-resistance (*aph*) genes serve as selective markers. Replication and copy number control are dependent on either the high copy number control pMB1*ori*, the medium copy *pir*-dependent *ori*R6Kγ or the single copy number maintenance *oriS* in combination with the inducible high copy number maintenance *oriV*-TrfA system (Metcalf et al., 1997; Zhang et al., 2000; Pritchett et al., 2004; Guss et al., 2008).

## Forward genetics

A transposon system derived from the *mariner* transposable element H*imar1*, which transposes to random sites in the genome at high frequency, is available for mutagenesis in *M. acetivorans* C2A (Zhang et al., 2000). The transposition of *mariner* elements is solely dependent on their cognate transposases and does not require host factors, hence they can be used for *in vivo* mutagenesis of a wide variety of eukaryotic and prokaryotic organisms (Lampe et al., 1996; Plasterk, 1996; Tosi and Beverley, 2000). The modified mini-H*imar1* transposon carries elements for selection in *Methanosarcina* (*pac* cassette) and in *E. coli* (*aph*) as well as the *ori*R6Kγ, for identification of the mutated gene through cloning of the insertion in *E. coli*. A suicide vector serves as the delivery plasmid. The *mariner* transposase (*tnp*) gene, controlled by the p*mcrB*, is not part of the transposable element, but resides on the plasmid and therefore perishes with the vector. This construct assures the stability of the insert, since the Tnp mediated transposition is fully reversible (Zhang et al., 2000).

## Reverse genetics

Integration of a cloned DNA fragment into the chromosome of *Methanosarcina* through homologous recombination was first achieved by Conway De Macario et al. (1996). Gene replacement or disruption can be facilitated by simply introducing the linearized cloning vector pBluescript (Stratagene) containing the appropriate homologous *Methanosarcina* DNA fragment and selective marker (Rother et al., 2005). As described above, only two selective markers, the *pac* and *ileS12* genes, are available for *Methanosarcina*. Thus, markerless deletions are preferred, because this allows repetitive use of both markers.

The first unmarked mutant constructed was a *M. acetivorans* C2A Δ*hpt* strain that served as the parent for subsequent deletion strains, since the loss of a functional *hpt* gene provides a convenient counter selective system (Pritchett et al., 2004). This principle was extended in a series of *M. acetivorans* C2A and *M*. *bakeri* Fusaro strains engineered to harbor a highly efficient site-specific recombination system at the Δ*hpt* locus that allows integration of genes into the chromosome (Guss et al., 2008).

**Figure 1:**
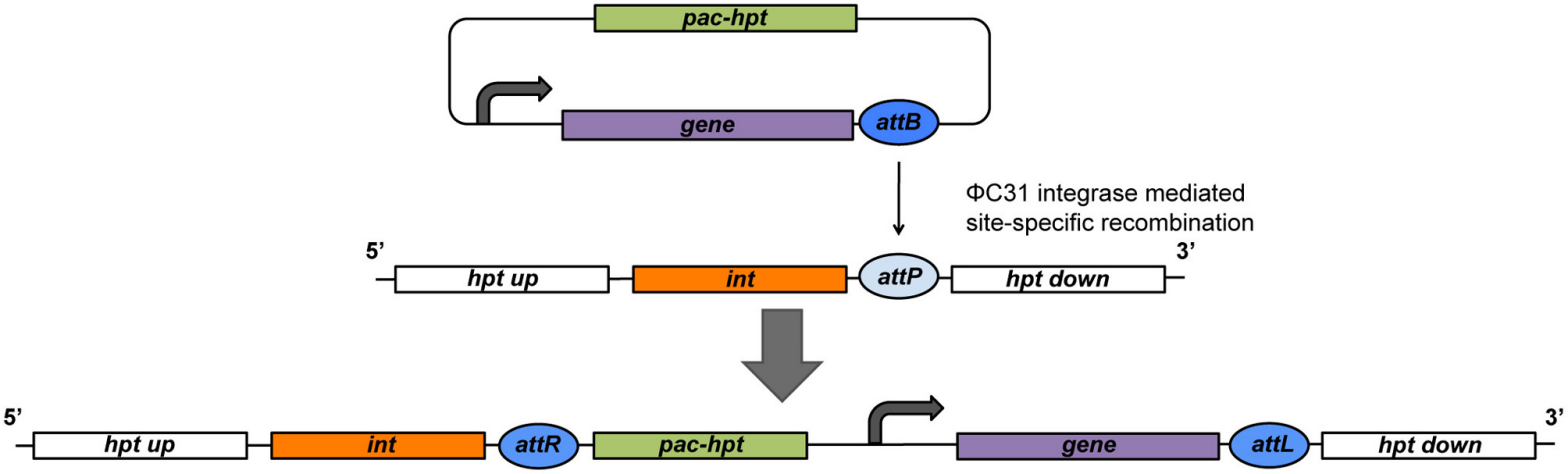
**Schematic of a site-specific recombination event at the engineered Δ*hpt* locus of the *Methanosarcina* chromosome.** The ΦC31 integrase (*int*) is constitutively expressed at the Δ*hpt* locus that also harbors a ΦC31 attachment site (*attB* in this figure). The integration plasmid contains the gene of interest controlled by a p*mcrB* derivative (curved arrow) and a ΦC31 attachment site (*attP* in this figure). *pac*, gene for puromycin *N*-acetyltransferase; *hpt*, gene for hypoxanthine phosphoribosyltransferase.

This system utilizes the host factor independent ΦC31 integrase of the *Streptomyces* bacteriophage ΦC31, encoded by the *int* gene, and the ΦC31 phage integration sites (Thorpe and Smith, 1998; Guss et al., 2008). The Int protein catalyzes site-specific recombination between the ΦC31 site at the Δ*hpt* locus (*attB* or *attP*) and the corresponding ΦC31 (*attP* or *attB*) of a plasmid (Figure 1). The reaction is unidirectional and results in the stabile integration of the entire plasmid. This allows the constitutive expression of the ΦC31 integrase from p*mcrB* without destabilizing the insert. Some constructs contain an artificial *tetR*-*int* operon, expressed from p*mcrB* (Guss et al., 2008). In general, the *tetR* gene codes for the TetR transcriptional repressor that binds the *tetO* operator of the target promoter and prevents transcription. TetR releases the promoter upon binding tetracycline and gene expression is initiated. This tetracycline regulated promoter system allows the tight regulation of cloned genes and is used for different applications in *Methanosarcina* (see below; Beck et al., 1982; Guss et al., 2008).

## Markerless exchange

Two methods, employing different mechanisms, have been established for routine use to generate unmarked mutants in *Methanosarcina* in the Δ*hpt* background (Pritchett et al., 2004; Welander and Metcalf, 2008). Both methods rely on homologous recombination, using deletion constructs consisting of either the 5′- and 3′- sequences of the gene, resulting in gene disruption, or the flanking up- and downstream sequences, resulting in gene deletion (Pritchett et al., 2004; Welander and Metcalf, 2008).

**Figure 2:**
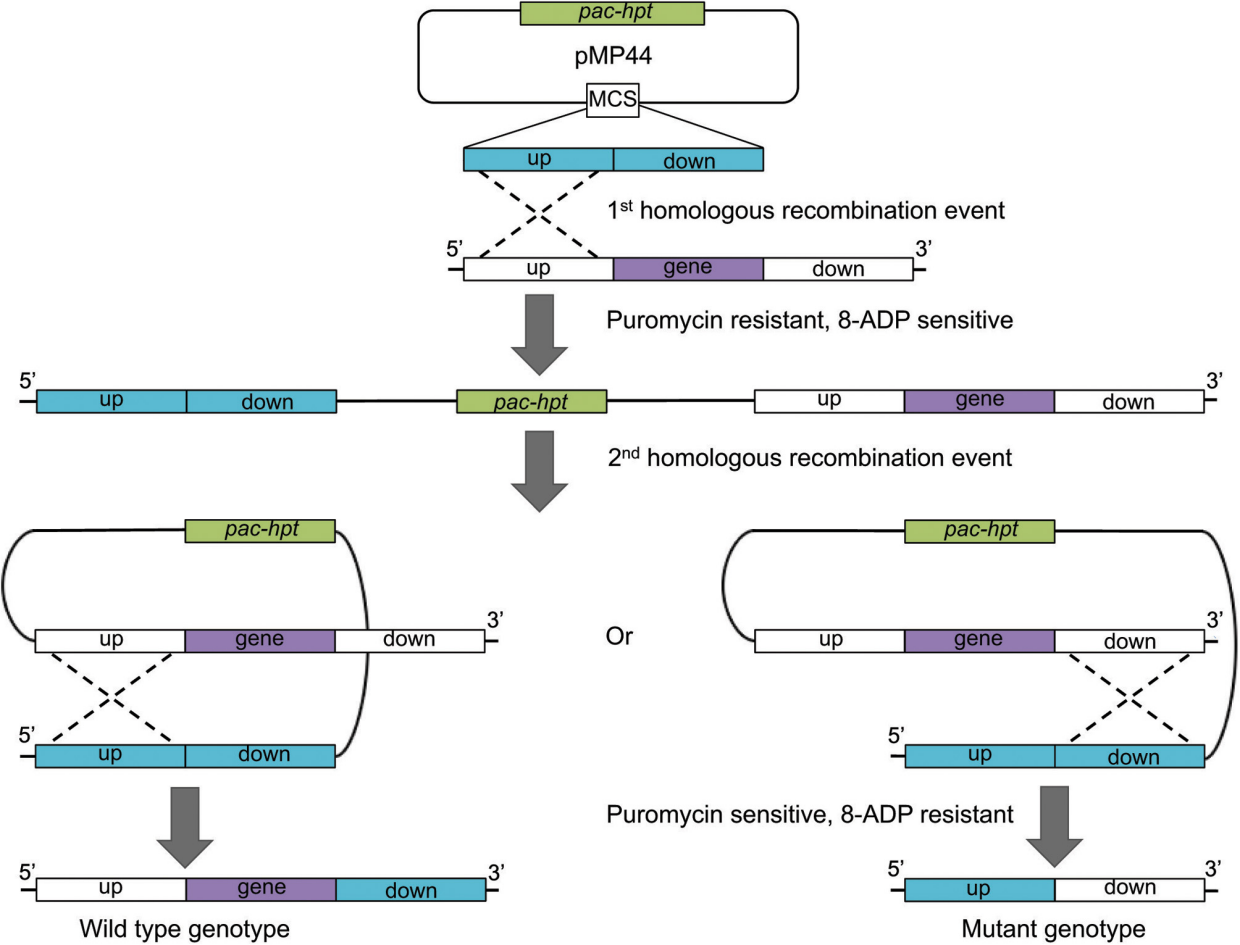
**Schematic of the construction of an unmarked deletion mutant strain using pMP44.** Plasmid pMP44 carries a deletion construct comprising the homologous up- and downstream regions of the target gene. The circular plasmid is transformed into a *Methanosarcina* Δ *hpt* strain. A homologous recombination event (dotted line) creates a puromycin resistant and 8-aza-2,6-diaminopurine (8-ADP) sensitive merodiploid intermediate. The resolution of the instable intermediate either reconstitutes the wild type genotype or results in the deletion of the target gene, both events restore puromycin sensitivity and 8-ADP resistance. *pac*, gene for puromycin *N*-acetyltransferase; *hpt*, gene for hypoxanthine phosphoribosyltransferase.

The first method makes use of the *pac* selectable marker and *hpt* counter selectable marker and relies on an unstable merodiploid intermediate state (Figure 2; Pritchett et al., 2004). Plasmid pMP44 does not replicate in *Methanosarcina* and carries the *hpt* gene in addition to the *pac* cassette. Derivatives of pMP44 containing a deletion construct are used to transform the *Methanosarcina* Δ*hpt* parental strain to puromycin resistance and 8ADP sensitivity during the first recombination event. The plasmid integrates into the chromosome at either the up- or downstream homologous regions, resulting in an unstable merodiploid, which is resolved by a successive second recombination event in the absence of selective pressure. This event removes the vector backbone including the *pac* and *hpt* genes and renders the progeny puromycin sensitive and 8ADP resistant (Figure 2). The second step gives rise to two types of 8ADP-resistant progeny, half the offspring will retain the desired mutation and the other half will retain the wild type locus, provided that the target gene is not essential and there is no difference in growth rate between the mutant and the wild type strain. Mutants are then identified via PCR screening or phenotypic testing.

**Figure 3:**
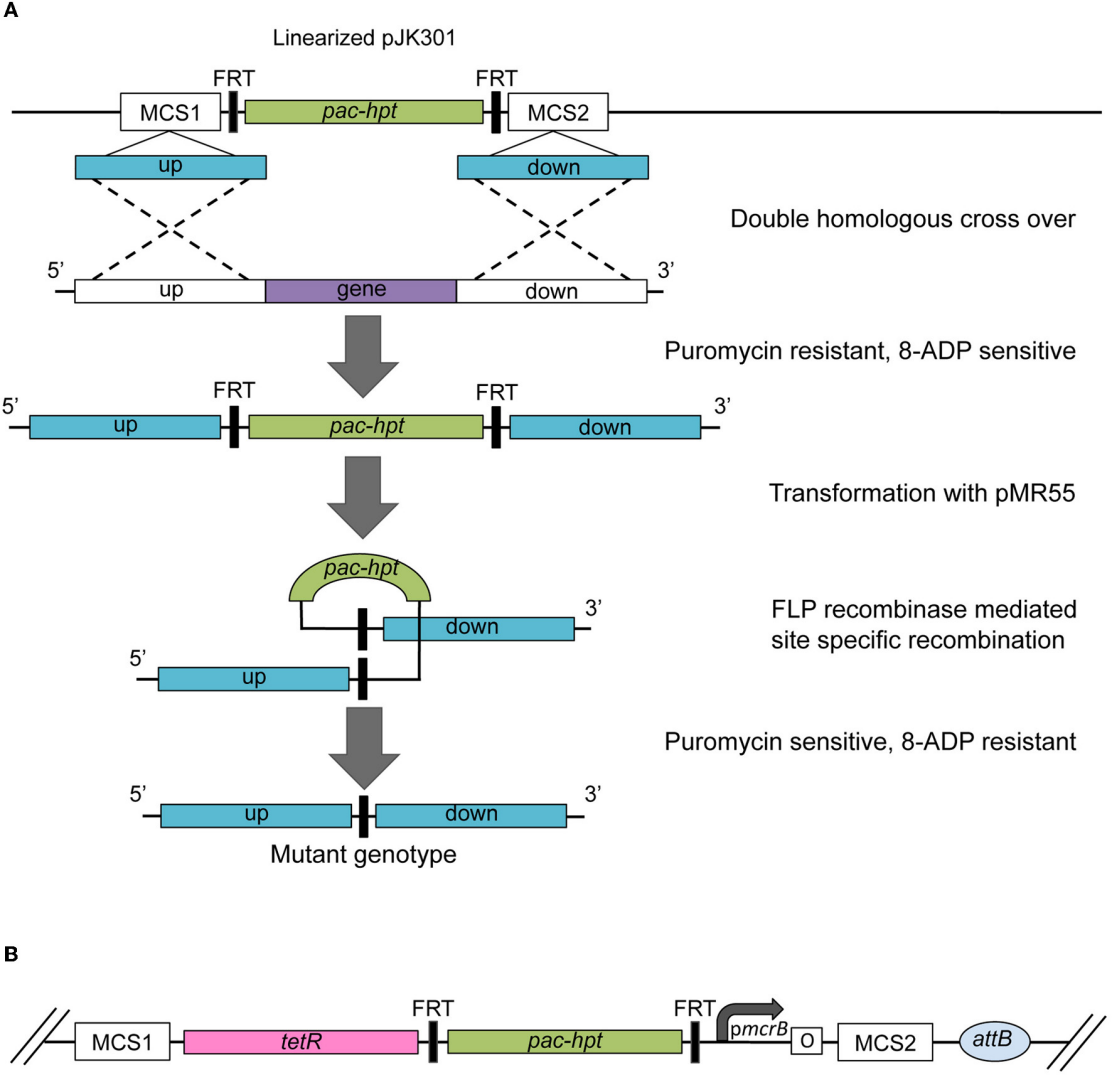
**(A)** Schematic of the construction of an unmarked deletion mutant strain using pMPJK301 and pMR55. The *pac*-*hpt* cassette of plasmid pMJK301 is flanked by the homologous up- and downstream regions of the target gene. The linearized plasmid is transformed into a *Methanosarcina* Δ*hpt* strain. A double homologous recombination event (dotted line) creates a puromycin resistant and 8-aza-2,6-diaminopurine (8-ADP) marked deletion mutant. Successive transformation with plasmid pMR55 results in the removal of the *pac*-*hpt* cassette via site-specific recombination between the FRT sites mediated by the Flp recombinase expressed from pMR55. The resulting mutant strain is puromycin sensitive and 8-ADP resistant. **(B)** Schematic of a plasmid used for promoter swaps. The p*mcrB* is represented by a bend arrow and the Tet operator by the letter “o” enclosed in a box. *pac*, gene for puromycin *N*-acetyltransferase; *hpt*, gene for hypoxanthine phosphoribosyltransferase; *tetR*, gene for the TetR transcriptional repressor.

The second established method is based on creating a marked mutant that allows subsequent removal of the selective and counter selective marker (Figure 3A; Rother and Metcalf, 2005; Welander and Metcalf, 2008). Plasmid pJK301 carries an artificial *pac-hpt* operon, flanked by two Flp recombinase recognition sites (FRT). Two multi cloning sites for the cloning of the desired homologous regions are located directly up- and downstream of the FRT-*pac*-*hpt*-FRT cassette. The plasmid, carrying the deletion construct needs to be linearized before transformation to ensure that the wild type gene is replaced or disrupted with the FRT-*pac*-*hpt*-FRT cassette through a double-recombination event. The resulting marked mutant is puromycin resistant and 8ADP sensitive. Introducing plasmid pMR55 that expresses the *Saccharomyces* Flp recombinase creates an unmarked, puromycin sensitive and 8ADP resistant mutant. The Flp recombinase recognizes the FRT sites and excises the *pac*-*hpt* cassette, leaving behind a “FRT scar.” The Flp recombinase system is highly effective, but fully reversible (Huang et al., 1997; Schweizer, 2003). Thus, the *flp* gene is transiently expressed and pMR55 does not replicate in *Methanosarcina*, in order to stabilize the construct.

## Genotypic complementation and reporter gene fusions

The respective wild type gene(s) can be reintroduced into *Methanosarcina* mutants through either plasmid based complementation or single integration into the chromosome.

## Genotypic complementation via multicopy plasmids and single copy integration

Multicopy expression can be achieved by using the *E. coli*/*Methanosarcina* shuttle vector pWM321 (Metcalf et al., 1997; Zhang et al., 2002). The plasmid offers a large multi cloning site but no promoter to drive the expression of the gene of interest and no RBS. A promoter-RBS-gene fusion needs to be cloned into pWM321. This provides the opportunity to tailor the construct to whatever is required by either choosing the native promoter of the gene, a promoter of known strength, or a tetracycline regulated promoter. The desired gene can either be cloned with its native RBS or placed under the control of the RBS of the methyl-reductase operon from *M. barkeri* Fusaro (p*mcrB* RBS).

## Genotypic complementation through single copy integration

A number of plasmids were designed to express genes of interest from either a constitutive or tetracycline regulated promoter (Guss et al., 2008). The p*mcrB* promoter achieves the highest level of constitutive expression. Lower constitutive expression levels can be obtained from the tetracycline regulated promoters, p*mcrB*(tetO1), p*mcrB*(tetO3), and p*mcrB*(tetO4), which decrease in promoter strength, respectively; if the plasmids are introduced into a host that does not express the *tetR* gene from the Δ*hpt* locus. All plasmids provide the p*mcrB* RBS and the p*mcrB* terminator and carry the FRT-*pac-hpt*-FRT cassette as well as a ΦC31 phage integration site. They do not replicate in *Methanosarcina* but integrate at the Δ*hpt* locus through site-specific recombination. Mutants that have successfully inserted the desired plasmid into the chromosome can be identified based on puromycin resistance and 8ADP sensitivity.

Another useful feature of the single copy expression vectors is one of the λ attachment sites (λ *attA* or λ *attB*) that can be used for retrofitting with other plasmids via a commercially available λ integrase system (Guss et al., 2008). This system can be used to turn the single copy expression vectors into autonomous, multicopy *Methanosarcina* plasmids through site-specific recombination with plasmid pAMG40 that carries the pC2A replicon. A *Methanosarcina* Δ*hpt* strain lacking the *int* gene and the ΦC31 integration site needs to be used as host for the expression plasmid:pAMG40 constructs to avoid integration into the chromosome.

## Gene expression studies, testing for gene essentiality and promoter swap

### Reporter gene fusions

**Figure 4:**
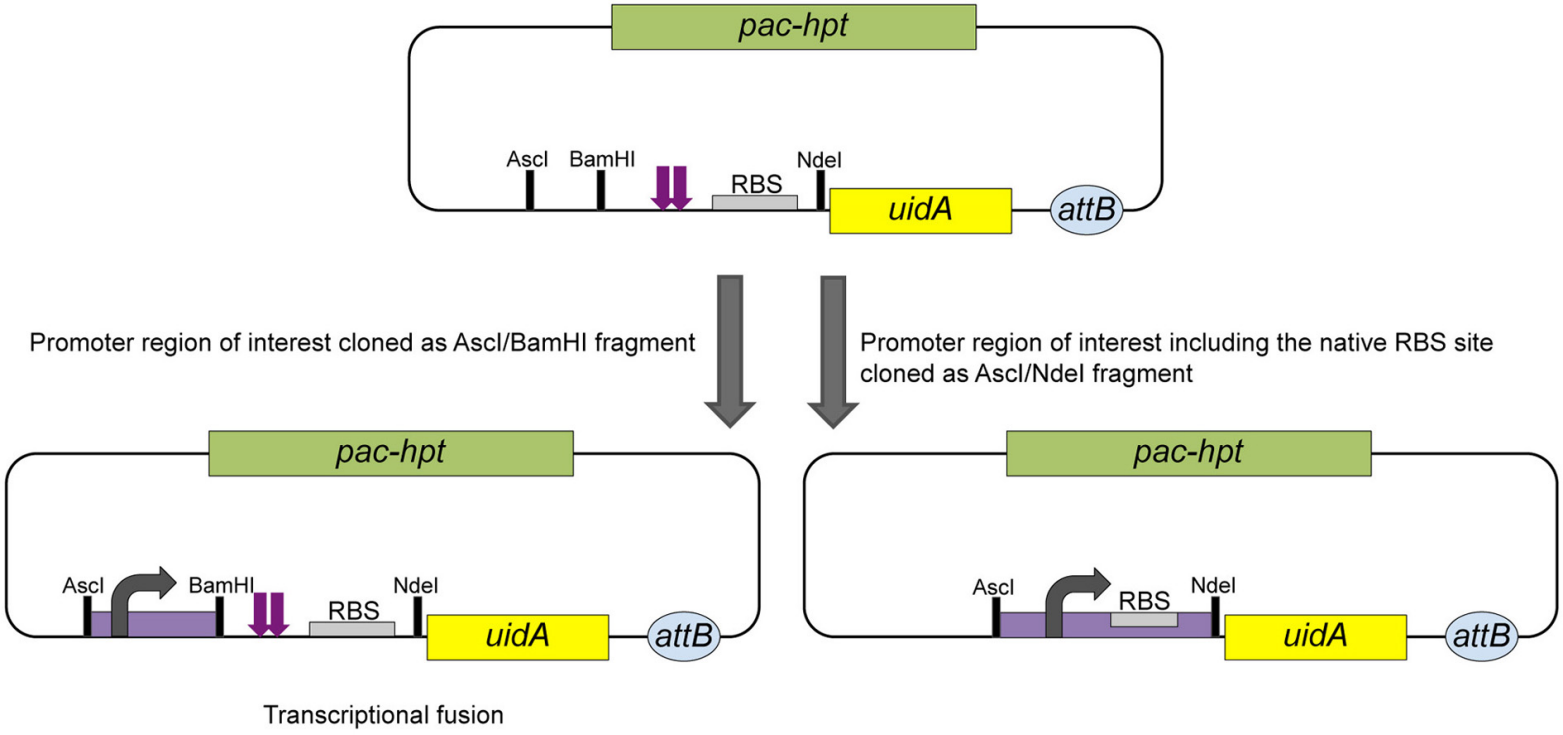
**Schematic of the construction of transcriptional and translational reporter gene fusions using pAB79.** Transcriptional fusions can be achieved by cloning the promoter region of interest as an AscI/BamHI fragment into pAB79. In this construct transcription is initiated from the cloned promoter (bend arrow). During translation no read through can occur from the cloned fragment into the uidA gene (β-glucuronidase) due the to tandem stopcodons (purple arrows). Translation of the β-glucuronidase is initiated from the p*mcrB* RBS (gray box). To create a translational fusion the promoter of interest, including its translational elements, is cloned as an AscI/NdeI fragment. *pac*, gene for puromycin *N*-acetyltransferase; *hpt*, gene for hypoxanthine phosphoribosyltransferase; *attB*, ΦC31 attachment site.

The widely used reporter gene *uidA*, encoding the *β*-glucu-ronidase from *E. coli*, is functional in *Methanosarcina*. Plasmid pAB79 was designed to allow the construction of transcriptional or translational fusions to *uidA* (Figure 4). Stable single copy fusions are advantageous for expression studies; pAB79 derivatives therefore integrate into the chromosome at the Δ*hpt* locus via ΦC31 mediated recombination (Guss et al., 2008).

For a transcriptional fusion the (putative) promoter of interest is cloned as an AscI/BamH1 fragment immediately upstream of tandem translational stop codons. The stop codons are followed by the further downstream located p*mcrB* RBS and *uidA* gene. In this construct transcription initiation is dependent on the cloned fragment, but translation initiation of the β-glucuronidase is based on the strong and effective p*mcrB* RBS provided by pAB79. The tandem stop codons guarantee no translational read through from the cloned fragment into the *uidA* gene (Figure 4).

To create a translational fusion the desired promoter is cloned as an AscI/NdeI fragment and replaces the tandem stop codons and the p*mcrB* RBS completely. The promoter's native RBS and other translational elements are maintained by converting the start codon of the respective gene into the NdeI site used for cloning. Transcriptional and translational initiation are dependent on the elements provided by the cloned fragment in this construct (Figure 4; Guss et al., 2008).

### Testing for gene essentiality and promoter swap

The methods for gene deletion discussed in the preceding sections cannot be employed to study the function of essential genes. Instead, a promoter swap can be used to create a conditional mutant as a way to investigate gene essentiality. In this method, the native promoter of the target gene is replaced with one of the tetracycline regulated promoters. A series of plasmids is available that are similar in design to the deletion vector pJK301 that carry the FRT-*pac*-*hpt*-FRT cassette for selection (Figure 3B; Guss et al., 2008). A *tetR* cassette is located upstream and a tetracycline dependent promoter downstream of the marker cassette. Two multi cloning sites flank this construct allowing the subsequent cloning of the upstream homologous region of the targeted promoter and the 5′ homologous region of the gene of interest. The promoter swap relies on the same mechanisms as the construction of a deletion mutant using the pJK301/pMR55 system (Figure 3A). If the targeted gene is essential the resulting mutant strain will only be viable in the presence of tetracycline.

## Conclusions and future directions

The availability of the genetic tools described above has greatly improved our knowledge of the methanogenic process in *Methanosarcina* species. Recent genetic studies have provided a better understanding of the C1 oxidation/reduction pathway and the energy-conserving electron transport chain of *M. bakeri* Fusaro (Welander and Metcalf, 2008; Kulkarni et al., 2009). It was also possible to characterize the biological roles of isozymes involved in different methanogenic pathways of *M. acetivorans* C2A, such as methanol and methylamine specific methyltransferases as well as cytoplasmic and membrane-bound heterosulfide reductases (Bose et al., 2009; Buan and Metcalf, 2010). The hydrogenases required for hydrogenotrophic methanogenesis are conserved in *M. bakeri* Fusaro and *M. acetivorans* C2A, cis-acting mutations were identified in the promoter regions of the respective genes of the latter that prevent gene expression and render the strain incapable of using *H*_2_/*CO*_2_ as growth substrates (Galagan et al., 2002; Guss et al., 2009). The ability to genetically manipulate *Methanosarcina* has not only contributed to a deeper understanding of methanogenesis, but has paved the way for the study of other cellular and metabolic functions such as transcriptional and post-transcriptional gene regulation (Bose et al., 2006, 2009; Bose and Metcalf, 2008; Opulencia et al., 2009) and mechanisms for synthesis and insertion of the 22nd genetically encoded amino acid, pyrrolysine, into methylamine methyltransferases of *Methanosarcina* (Mahapatra et al., 2006, 2007).

Ongoing and future research employing genetic methods in combination with the classical physiological and biochemical approaches will undoubtedly expand our understanding of methanogenic archaea even further. In addition, the ability to genetically manipulate *Methanosarcina* spp. opens up the possibility to metabolically engineer strains for the production of biogas from organic materials. Methane, one of the major end products of methanogenesis, represents a clean burning and renewable energy source. *Methanosarcina* spp. are among the most promising candidates for routine use in biofuel production, because among the methanogenic archaea *Methanosarcina* have the broadest range in substrates for methanogenesis and exhibit the highest tolerance to environmental stresses (De Vrieze et al., 2012). Engineering efforts to further enhance stress tolerance, optimize the efficiency of substrate usage and broaden the substrate range of *Methanosarcina* spp. could significantly improve their performance in industrial settings like anaerobic digesters, and optimize methane yields. For example, the introduction of a gene coding for a broad-specificity esterase from *Pseudomonas veronii* into *M. acetivornas* has resulted in a strain that efficiently converts methyl acetate and methyl propionate to methane (Lessner et al., 2010).

### Conflict of interest statement

The authors declare that the research was conducted in the absence of any commercial or financial relationships that could be construed as a potential conflict of interest.
